# A Comparative Study between Indigenous Low Cost Negative Pressure Wound Therapy with Added Local Oxygen versus Conventional Negative Pressure Wound Therapy

**DOI:** 10.5704/MOJ.2011.020

**Published:** 2020-11

**Authors:** A Singh, K Panda, J Mishra, A Dash

**Affiliations:** Department of Orthopaedics, Siksha O Anusandhan University Institute of Medical Sciences and SUM Hospital, Bhubaneswar, India

**Keywords:** negative pressure wound therapy, vacuum assisted closure, NPWT, VAC, compound fracture

## Abstract

**Introduction:**

The incidence of compound fractures and severe soft tissue loss has increased manifolds due to high speed traffics. Negative Pressure Wound Therapy (NPWT) is a treatment modality for managing soft tissue aspect of such injuries. It reduces the need of flap coverage. However, many patients from developing countries cannot afford a conventional NPWT. We developed an indigenous low cost NPWT for our patients and supplemented it with Topical Pressurised Oxygen Therapy (TPOT). We conducted this study to compare its treatment outcome with the use of conventional NPWT.

**Materials and Methods:**

The study was conducted from 2018 to 2020 at a tertiary care teaching hospital. A total of 86 patients were treated with NPWT and their results were assessed for various parameters like reduction in wound size, discharge, infection, etc. We included patients with acute traumatic wounds as well as chronic infected wounds, and placed them in three treatment groups to receive either conventional NPWT, Indigenous NPWT and lastly NPWT with supplement TPOT.

**Results:**

We observed a significant reduction of wound size, discharge and infection control in all three groups. The efficacy of indigenous NPWT is at par with conventional NPWT. Only six patients who had several comorbidities required flap coverage while in another four patients we could not achieve desired result due to technical limitations.

**Conclusion:**

Indigenous NPWT with added TPOT is a very potent and cost effective method to control infection and rapid management of severe trauma seen in orthopaedic practice. It also decreases the dependency on plastic surgeons for management of such wounds.

## Introduction

Negative pressure wound therapy (NPWT) is a time tested treatment modality in management of compound injuries where primary closure could not be achieved. NPWT was introduced in clinical practice in the 90’s and has since been widely used^1^. In 1992, in Germany, the patients with exposed fractures were treated with a negative pressure system. Argenta and Morykwas extensively studied NPWT and its effects^2-4^. The different effects of NPWT as described by various studies are reduction in wound size^2-4^, stimulation of granulation tissue^2^, removal of small tissue debris, decreased protease content, and removal of exudate. It also reduces the interstitial edema^2,3,5^ thereby increasing microcirculation, local blood flow and oxygenation^5^. It has been shown to promote angiogenesis and increase the level of Vascular Endothelial Growth Factor (VEGF)^2,6^. Moreover, it has a special role in management of infected wounds with heavy exudate that warrants regular dressing change. Frequent change of dressings and prolonged hospitalisation may increase the financial burden for the patient as well as the healthcare system. NPWT which is more commonly known as vacuum assisted closure (VAC) helps to combat this situations as it decreases the frequency of dressing change and days of hospitalisation.

VAC or NPWT works by creating a closed environment under negative pressure where all the exudate and local oedema is sucked out of the wound, and growth of granulation tissue is enhanced. There is also increased extravascular migration of neutrophils and macrophages that helps in phagocytosis of bacteria. It also decreases the bacterial load and creates a favourable hypoxic environment in initial stages of neo-vascularisation. These all lead to a decrease in wound dimensions like length, breadth and depth, so that ultimately it can be closed with secondary suturing or covered with a skin graft. Many a times the depth of the wound is decreased to such an extent that spontaneous epithelisation occurs and no definite procedure is required.

It is a known fact that a healing skin has more metabolic demands as compared to intact skin, therefore more oxygen is required to meet these demands. In the different phases of wound healing, numerous biochemical and cellular processes depend on oxygen supply^7,8^. Generation of Reactive Oxygen Species (ROS), infection control, extracellular matrix formation and remodelling of collagen all require oxygen^9-11^. Various methods has been tried to provide additional oxygen to the wounds through either hyperbaric/normobaric oxygen therapy.

Only a small amount of oxygen can reach the tissue topically as the fluids present in the wound bed acts as barrier^12^. Blackman *et al* reported that by using Topical Pressurised Oxygen Therapy (TPOT) the wounds are more likely to heal faster^13^. Gordillo *et al* reported more rapid reduction in wound size along and increased VEGF expression in the wound with TPOT^14^. Gordillo and Sen recommended that it should be used for 90 minutes daily for four consecutive days followed by three days without treatment^15^.

There are various methods of applying VAC therapy. Conventional VAC therapy uses an automated microprocessor controller placed in an attached canister. The rental of this apparatus is expensive and many patients may not be able to afford it. To make it available for the general population, we developed an indigenous way provide VAC therapy. With this method of VAC, we can also apply TPOT to the wound. The apparatus can be used in any type of setup, and can be fabricated with readily available materials without the use of sophisticated machines. We decided to study the efficacy of this indigenous VAC therapy with and without TPOT, and compare it with the use of conventional VAC.

## Materials and Methods

**Fig. 1:**
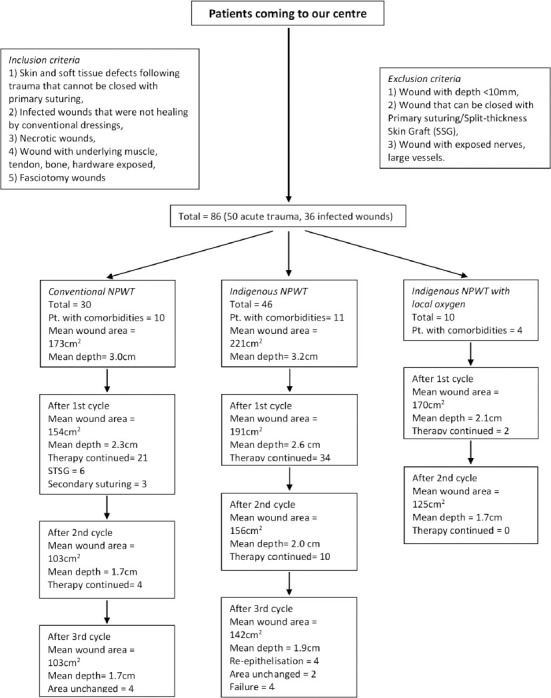
Randomised controlled study design and fate of all three treatment arms

Permission was obtained from the Institutional Ethics Committee before starting the study. The study was conducted in a tertiary care hospital in Eastern part of India from December 2018 to March 2020 on eighty six patients out of which fifty eight were males and the rest were females. Inclusion criteria for our study were: 1) skin and soft tissue defects following trauma that cannot be closed with primary suturing, 2) infected wounds that were not healing by conventional dressings, 3) necrotic wounds, 4) wound with underlying muscle, tendon, bone, hardware exposed, and 5) fasciotomy wounds. The exclusion criteria were 1) wound with depth <10mm, 2) wound that can be closed with primary suturing/split-thickness skin graft (SSG), and 3) wound with exposed nerves, large vessels (Fig. 1).

The patients getting admitted to our hospital were selected on the basis of inclusion and exclusion criteria. Informed written consent was taken from all the patients. Patients who could afford conventional NPWT were given that treatment while those could not afford it were given the indigenous low-cost NPWT. Among the patients treated with indigenous NPWT, a few randomly selected patients were offered hyperbaric oxygen therapy (Fig. 1). The statistical analysis of data was performed using the Statistical Package for Social Sciences [SPSS for Windows, version 20.0. Chicago, SPSS Inc] and Microsoft Excel 2010.

Fifty patients presented with acute trauma (<72 hours). Some of them sustained fractures or dislocations that had been treated with either internal or external fixations. There were also injuries that were complicated with compartment syndrome and had undergone fasciotomy. Thirty-six patients had infected wounds after trauma, surgery, or chronic osteomyelitis, and had been treated with conventional dressings.

**Fig. 2:**
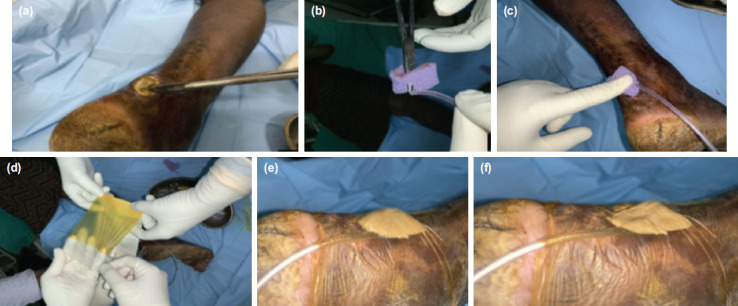
Steps of application of Indigenous NPWT. (a) Sharp debridement. (b) Measurement and trimming of foam. (c) Placement of foam and Infant feeding tube. (d) Airtight sealing with a piece of IOBAN (3M). (e) Before negative pressure application. (f) Wrinkling and shrinkage after negative pressure application. (NPWT : Negative Pressure Wound Therapy. IOBAN : Iodine impregnated Antimicrobial Incise Drape by 3M).

After taking blood samples for investigation, wound swabs were sent for bacterial culture and antibiotic sensitivity testing. The wound was washed thoroughly with povidone iodine solution, normal saline and hydrogen peroxide. We would perform surgical debridement under aseptic conditions to the level of healthy looking tissue and bleeding wound margin (Fig. 2a). Underlying fracture (if required fixation) would be fixed at this stage. We record all relevant clinical parameters that include wound diameter (length, breadth and depth), underlying structures (muscle, fat, fascia, tendons, hardware, bone), nature of discharge, etc. Thirty patients were treated with conventional NPWT therapy, forty-six patients were treated with the indigenous NPWT therapy alone, and ten patients were treated with the indigenous NPWT alternating with TPOT (Table I). Patients with history of smoking and diabetes mellitus were distributed equally in all groups to eliminate bias due to confounding factors.

**Table I:** Patient data prior to application of NPWT

	Conventional VAC	Indigenous Low Cost VAC	Low with	Cost VAC TPOT
No. of Patients	30	46		10
Mean Area (cm2)	173	221		210
Mean Depth (cm)	3.0	3.2		2.9
Positive cultures	19	29		7
Mean exudate (ml/day)	186	201		175
Smoking or Diabetes Mellitus History	10	11		4
Exposed Bone / Hardware / Tendon	11	14		5

For the indigenous NPWT we used commercially available open cell polyurethane foam. It was sterilised using ethylene oxide at our hospital’s central sterile supply department. This foam was cut to appropriate size that corresponded to the dimensions of the wound, avoiding extension beyond the wound margins (Fig. 2c). We insert the distal fenestrated end of an infant feeding tube (Fig. 2b) inside the foam by making a tunnel in it. Additional holes can be made in the distal part of feeding tube if the wound size is large. This was placed over the wound and covered with an antimicrobial drape [IOBAN, 3M, USA] making sure it covers at least 2-3cm of surrounding skin to provide air tight seal (Fig. 2d). Alternatively, a sterile latex glove can also be used at sites where application of antimicrobial drape is difficult like distal part of foot where web spaces are also involved.

The airtight seal was confirmed by creating a negative pressure with 50ml syringe, and looking for wrinkling and contraction over the surface of the drape (Fig. 2e and 2f). The proximal end of feeding tube was then connected to a suction catheter (FG 12/14) which was connected to a vacuum pressure gauge with the help of sterile rubber tubes. The pressure was set between -125 to -150mmHg^2,3,16,17^ and applied continuously for first 24 hours. Subsequently it can be set to two hours on and one hour off (negative pressure maintained for two hours and released for one hour) for the next 72 hours. Ten patients received TPOP after the first 24 hours using the same apparatus/tubing. Only the source was changed to oxygen supply line and set to 3 L/min for 90 minutes a day^15^.

We provided empirical antibiotics (with anaerobic coverage) during the initial period and subsequently converting to definitive antibiotics based on culture and sensitivity reports. The wound exudate which was collected in vacuum jar was examined daily for its quantity and colour. The dressings were removed after 96 hours of initial application and was the wound was examined for its dimensions, appearance, presence of slough, discharge, etc. Wound swabs were again taken for culture and sensitivity testing. If required, more cycles of NPWT therapy would be provided. The end point of NPWT dressing were any one of the following; 1) depth <10mm, 2) wound edges can be closed by secondary suturing, and 3) wound ready for SSG. We would consider the treatment as failed in the following conditions ; 1) wound dimensions increased (even if due to re-debridement), 2) wound infection and discharge worsening over time, 3) wound diameter unchanged after application of 2 VAC cycles, and 4) infection / necrosis spreads to surrounding area. SSG was also done in our department and signs of graft take up and rejection were recorded.

## Results

**Fig. 3:**
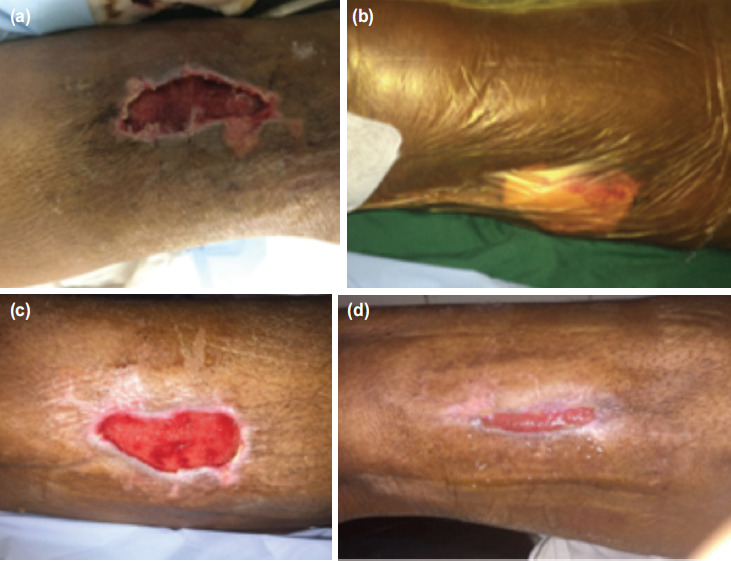
(a,b,c,d) A patient of Morel – Lavallee Lesion at lateral aspect of thigh treated with Indigenous NPWT (3 cycles- 14 days) and healed with secondary intention.

**Fig. 4:**
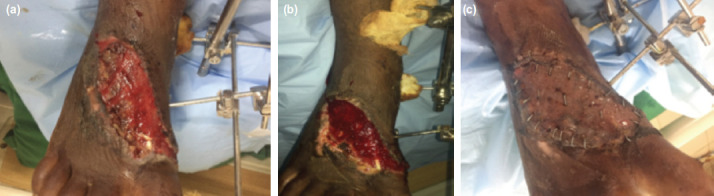
Patient with wound over dorsal aspect of right foot and external fixator applied to span the ankle joint and immobilisation, a) after 1 cycle of indigenous NPWT+TPOT. (b) Wound after two cycles of Indigenous NPWT+TPOT. (c) Post STSG application.

Mean wound area and depth reduction was 35.4% and 38.9% for indigenous NPWT; 39.3%, 40.4% for indigenous NPWT with oxygen therapy; and 40.2%, 42.3% for conventional NPWT (Table II and III). In case of previously infected wounds, sterile cultures were obtained in 72% (21/29) of patients using indigenous NPWT, 79% (15/19) of patients using conventional NPWT and 85% (6/7) of patients using indigenous NPWT with TPOT group after completion of therapy. For patients who used conventional NPWT, 78.3% (36/46) were ready for definitive procedures after 1-2 cycles (4-8 days) of treatment. Twenty-six of them had SSG and ten had secondary suturing. For patients who used the indigenous NPWT, 8.7% (4/46) of them had drastic reduction of wound depth after 3 cycles of NPWT (Fig. 3), and subsequently their wounds developed re-epithelisation without any need of SSG (Table III). For patients who used the conventional NPWT, 86.7% (26/30) were ready for definitive procedure after 1-2 cycles of therapy, fourteen had SSG and twelve had secondary suturing. All the ten (100%) patients who used indigenous NPWT and TPOT proceed to definitive treatment (6 SSG and 4 secondary suturing) (Fig. 4) with one to two cycles of therapy (Table III).

**Table II:** Patient data after 1-3 cycles of NPWT

	Conventional VAC	Indigenous Low Cost VAC	Low Cost VAC with TPOT
No. of Patients	30	46	10
Mean Area (cm2)	103	142	125
Mean Depth (cm)	1.7	1.9	1.7
Positive cultures	4	8	1
Mean exudate (ml/day)	25	31	16
Exposed Bone / Hardware / Tendon	4	6	2

**Table III:** Results of NPWT

	Conventional VAC	Indigenous Low Cost VAC	Low Cost VAC with TPOT
No. of Patients	30	46	10
Mean Area reduction %	40.2	35.4	39.3
Mean Depth reduction %	42.3	38.9	40.4
Infection controlled %	79	72	85
Mean exudate reduction %	86.5	84.5	90.8
STSG	14	26	6
Secondary suturing	12	10	4
Healing with secondary intention	-	4	-

Two patients who used indigenous NPWT developed latex allergy and unhealthy surrounding skin during the first cycle of therapy, and we changed the covering to antimicrobial drape for next cycle. Two patients who had SSG following therapy using conventional NPWT had signs of graft rejection on the fifth post-operative day, and repeated grafting procedure healed uneventfully. In four patients treated with indigenous NPWT, the depth of the wounds was far greater than the width/length and they developed foam adherence with pocket of pus. They were treated with re-debridement and considered as treatment failures. Four patients (two using conventional NPWT and two using indigenous NPWT) who previously had infected wound did not obtain desired result because the wound areas remain unchanged with no reduction in amount of discharge. They were subsequently treated with rotation flaps by a plastic surgeon. (Table IV). Information on the mean wound sizes before and after the NPWT, and the final procedure performed in all three treatment arms is given in Fig. 1.

**Table IV:** Complications and Failures

	Conventional VAC	Indigenous Low Cost VAC	Low Cost VAC with TPOT
Final No. of Failures	4	6	0
Pain during dressing change	10	13	6
Skin maceration/ Latex allergy	0	2	0
Wound dimensions did not reduce	4	6	0
Bleeding during dressing change	5	7	4
STSG uptake problems	2	0	0

The results were evaluated using student t-test and there was no statistically significant difference in wound area and depth reduction between the three treatment arms (p>0.05).

## Discussion

We conducted a prospective control trial where patients with soft tissue trauma were treated with three different modalities of treatment. Our study contained patients who either had extensive soft tissue loss with compound fractures following trauma, or infected wound treated with conventional wound dressings. Most of the acute trauma patients were males in their third and fourth decades who were involved in motor vehicle accidents. Most patients who were in their sixth and seventh decades had infected wounds and co-morbidities like diabetes mellitus.

A study on open fractures showed that patients treated with NPWT were only one fifth as likely to have deep infection compared to those treated with standard fine mesh gauze dressing (relative risk of 0.199; 95% confidence interval: 0.045-0.874)^18^. Lee *et al* reported that NPWT applied for 1129 days over exposed tendon or bone resulted in healing by secondary intension in all but 1 patient out of 16 who required flap coverage^19^. Stannard *et al* reported that NPWT group showed significantly lower risk of infection compared to the control group with p-value of 0.024, with better control of infection and increased granulation tissue^18^. Sinha *et al* reported that over eight days the NPWT group showed significant wound size reduction of 13.24mm with a p-value of 0.000120.

Morykwas *et al*^2,4^ extensively studied negative pressure wound therapy and concluded that at pressures of - 125mmHg the microvascular blood flow increased to four times of its baseline value and it was inhibited at pressure levels equal to or lower than -400mmHg. Only a small amount of oxygen can enter the tissue topically as the fluids present in the wound bed acts as barrier^12^. To cope up with this we increased the pressure of topical oxygen therapy, which would increase the partial pressure and hence the dissolved oxygen. Other studies have demonstrated the beneficial effect of increased partial pressure of oxygen^21^. Several researchers have demonstrated improvement in wound healing by using topical pressurised oxygen therapy^22^. Improvement of local blood supply also increases the amount of dissolved oxygen^23^. In our study this was achieved by concomitant use of NPWT which increases local blood supply^5^.

In a randomised study by Egington *et al*, change in wound depth and volume were studied with application of NPWT for two weeks compared to conventional moist dressings^24^. In NPWT group the decrease in wound depth was 49% and decrease in volume was of 59%. Isago *et al* conducted a similar study and took variables like wound surface area and depth into consideration, and his results showed reduction of 55.1% and 61.2%, respectively^25^. Another study by Herscovici *et al* in 21 patients with high-energy trauma wounds treated with VAC noted lesser number of dressings and a decreased hospital stay as compared to those treated with conventional dressings^26^. He also concluded that there was a decreased requirement of flap coverage when VAC was applied early. Another study reported that NPWT lead to highest eradication rate for prosthetic infections^27^. One study suggested that NPWT resulted in significant reduction in deep wound cavity/defects^28^. Other studies suggest that NPWT is the treatment of choice when plastic surgery procedures cannot be used for coverage exposed bone, tendon or metalwork^29^.

We observed treatment failures in four patients with infected wounds where the depth was far greater than the wound opening. This is most likely due to early approximation of the superficial small wound that eventually closed, leaving a potential cavity that subsequently filled up with pus, and occasional foam adherence. This could have been avoided if we had extensively debrided the superficial part of the wound and made the opening larger and use of a higher density foam.

Our study showed that the indigenous NPWT can provide similar findings can significantly decrease the wound dimensions and reduce the number of dressings with lower cost. Only six patients (6.9%) patients required flap coverage. The other benefits were control of exudate, wound infection and odour. In 1995 the US Food and Drug Administration approved the use of VAC for non-healing ulcer management. Now, the indication for VAC is wide and includes but is not limited to chronic, acute, traumatic and subacute wounds, grafts and flaps. The contra-indication for application of VAC are high output wounds, underlying osteomyelitis, fistulas, exposed neuro-vascular structures, malignant wounds and dry gangrene.

## Conclusion

Our study showed that an indigenous NPWT which costs around 8 USD (INR 663) per cycle is equally efficacious as that of conventional NPWT which costs 100 to 200 USD (INR 7000 to 14000 per cycles). Addition of TPOT may further improve its outcome. Therefore, we can conclude that in resource limited settings, a low cost NPWT with or without TPOT can be used safely and effectively for management of extensive Musculo-skeletal trauma wounds.
